# Pediatric Uveitic Glaucoma

**DOI:** 10.5005/jp-journals-10008-1147

**Published:** 2013-09-06

**Authors:** Savleen Kaur, Sushmita Kaushik, Surinder Singh Pandav

**Affiliations:** Senior Resident, Glaucoma Services, Advanced Eye Center, Postgraduate Institute of Medical Education and Research, Chandigarh, India; Associate Professor, Glaucoma Services, Advanced Eye Center, Postgraduate Institute of Medical Education and Research, Chandigarh, India; Professor, Glaucoma Services, Advanced Eye Center, Postgraduate Institute of Medical Education and Research, Chandigarh, India

**Keywords:** Uveitic Glaucoma, Children, Intraocular pressure, Corticosteroids.

## Abstract

The pediatric glaucomas present some of the greatest clinical challenges. Uveitic glaucoma is a pathology whose causes and treatment are still a great enigma to all glaucoma specialists. We describe the spectrum of pediatric uveitic glaucoma along with its risk factors and the outcome of treatment in this subgroup. Our paper aims to review the existing literature on the subject and throw light on how to manage these cases.

**How to cite this article:** Kaur S, Kaushik S, Pandav SS. Pediatric Uveitic Glaucoma. J Current Glau Prac 2013;7(3): 115-117.

## INTRODUCTION

Glaucoma in childhood is a potentially blinding condition, yet, there is a paucity of epidemiologic and clinical data regarding this pathology. Uveitis is a cause of secondary glaucoma in children, but its severity depends on the type, duration of the disease, the ability to control the intraocular infammation as well as the corticosteroid therapy. How the pediatric eyes respond to pathologies and the subsequent treatment strategies, is largely dependent on early, accurate diagnosis and successful treatment involving intraocular pressure (IOP) control to a level where progression is unlikely, along with the prevention of amblyopia. The most common causes of vision loss in children with uveitis are cataract, band keratopathy, glaucoma and cystoid macular edema, making it a dangerous potentially blinding disorder. This review will look at the epidemiology, presentation and management options for uveitic glaucoma in children.

### Incidence

Pediatric uveitis and uveitic glaucoma are two entities which are grossly understudied. Children constitute 5 to 10% of the patients with uveitis seen at tertiary referral centers.^1^ As many as one third of these children are reportedly left with severely impaired vision due to the uveitic compli cations. Studies on pediatric glaucoma report the underlying cause as uveitis to be in between 6 to 9%.^2,3^ Uveitis contributed to 19% of the glaucoma among the 52 pediatric patients with glaucoma over a 1 year study group in the BIG eye study done in the United Kingdom.^4^ The prevalence of glaucoma in children with uveitis ranges between 5 and 13.5%.^5^ However, Paroli et al^6^ reported that 25% of children with uveitis developed secondary glaucoma.

**Fig.1:**
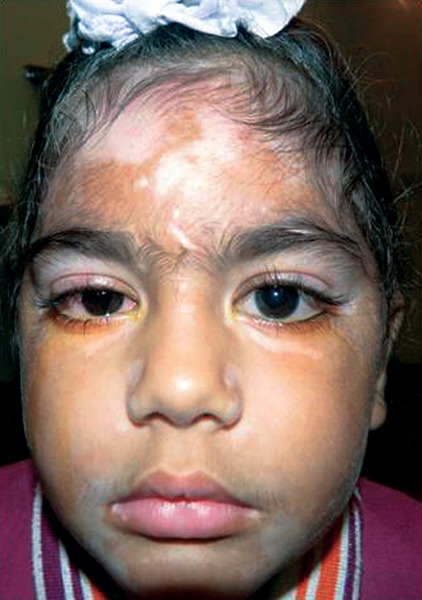
Uveitic glaucoma in a child with VKH syndrome (*Courtesy*: Dr Sushmita Kaushik, Chandigarh, India)

**Fig. 2:**
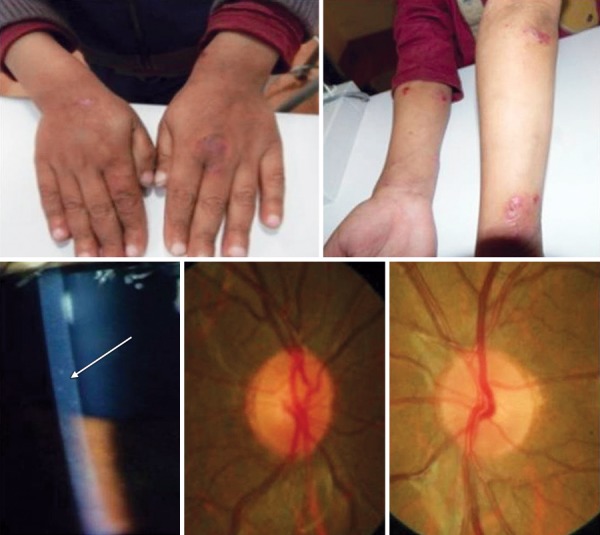
Uveitic glaucoma in a child with Wiskott-Aldrich syndrome (*Courtesy*: Dr Sushmita Kaushik, Chandigarh, India)

Unpublished data from our center looked at our incidence. Hospital records of patients attending the pediatric glaucoma clinic from July 2005 to December 2012 at our center were reviewed (unpublished data). Among 385 pediatric glaucoma patients, 150 children had acquired glaucoma of which eight patients had uveitis as the underlying cause (5.3%). Ten-year data from the uveitis clinic in our center (Ramandeep Singh, unpublished data) revealed 188 patients less than 16 years of age with uveitis. Of these, glaucoma was seen in six patients. Three had panuveitis, two had intermediate uveitis and one had anterior uveitis (Figs 1 and 2).

As a result of relative rarity of uveitic glaucoma and difficulty faced in examining children, uveitic glaucoma in the pediatric population is at risk of being misdiagnosed or suboptimally treated, allowing irreversible corneal and optic nerve damage to occur.

### Risk Factors

Pediatric eyes with uveitis have an inherent predisposition to develop glaucoma as compared to adults.^7^ The prevalence of glaucoma among uveitis patients also depends on the underlying disease and its duration.^8^ An epidemiologic study of pediatric glaucoma from Toronto^9^ reported glaucoma secondary to uveitis, aniridia, anterior segment developmental anomalies involving the cornea, and retinopathy of prematurity as being the groups with the worst visual outcomes. Juvenile idiopathic arthritis (JIA) has been reported to be particularly susceptible to develop glaucoma. It has been reported in 42 to 48% of patients with juvenile rheumatoid arthritis and these children have a poor visual outcome when they develop early onset glaucoma.^10-12^ However, in our cohort, JIA was not very common in pediatric uveitic cases seen in our center (unpublished data, Ramandeep Singh: 26 patients (13.8%) of the 188 patients had JIA). Other risk factors for glaucoma in the uveitis patient are aphakia and status after vitrectomy.

### Mechanism and Diagnosis

The mechanism of increased IOP is complex in patients with uveitis. Infammatory cells and corticosteroid therapy each can contribute to the pathogenesis of uveitic glaucoma. The glaucoma can be both open and closed angle. Angle closure can occur with extensive posterior synechiae resulting in pupil block or with peripheral anterior synechiae without pupil block. Open-angle glaucoma in uveitis can occur from obstruction of aqueous outfow as a result of steroid use or infammation induced trabecular obstruction, or trabeculitis. In chronic anterior uveitis, glaucoma is thought to result from trabecular scarring and/or sclerosis caused by chronic trabeculitis. Sometimes, it can be difficult to differentiate steroid-induced glaucoma from uveitic glaucoma. It is very important in these cases to establish a low to normal IOP when the uveitic attack has subsided and the patient is not on steroid treatment. It is equally important to document an initial low IOP in some cases before uveitis begun in pediatric eyes to actually call it a uveitic glaucoma. Nevertheless, there may be a large segment of uveitic patients with combined mechanism glaucoma due to the infammation as well as the use of corticosteroids. These patients pose clinically challenging cases to treat because there is no singular mechanism causing the glaucoma which can be treated.

### Treatment

As mentioned, the management of uveitic glaucoma may be difficult because of the numerous mechanisms involved in its pathogenesis. In addition, the hypotensive effect of some glaucoma medications, such as latanoprost and brimonidine, may be blocked partially by the concurrent administration of nonsteroidal anti-infammatory drugs.^13^ Prostaglandin analogs have a limited use in uveitis patients since they may exacerbate the infammation.

Uveitic glaucoma refractory to medical treatment needs to be surgically managed. Various surgical techniques have been described for controlling intractable glaucoma due to infammatory glaucoma in children and young adults. Trabeculectomy with adjutant antifibrotics is the time honored treatment in most of the cases of glaucoma requiring surgery. One study^14^ between mitomycin C trabeculectomies and Ahmed valves for complicated pediatric glaucoma including 14 eyes with uveitic glaucoma (three of which underwent Ahmed valve implantation and 11 mitomycin C trabeculectomy). The Ahmed valve group was twice as likely to use antiglaucoma medications postoperatively (44 *vs* 23%). The mitomycin C trabeculectomy group was more likely to have a 3-line decrease in Snellen visual acuity than the Ahmed valve group (28 *vs* 12%). Frequent postoperative infammation often leads to fltration surgery failure; thus glaucoma drainage devices appear to be more successful in uveitic glaucoma than in other recalcitrant types of glaucoma.^13^

Other surgical procedures have been tried in an attempt to increase the success rate. Freedman et al reported overall success rate of 75% using standard goniotomy surgery for the treatment of young patients with medically refractory glaucoma associated with chronic childhood uveitis with few complications and without worsening of existing uveitis.^15^ Trabeculodialysis, a modifed goniotomy procedure, has also been reported to be useful in the treatment of glaucoma associated with chronic uveitis.^16^

### Outcome in Uveitic Glaucoma

Despite aggressive medical and surgical therapy, uveitic glaucoma has been reportedly associated with progressive visual field loss and optic nerve damage in 33% of uveitis patients in a tertiary referral center.^17^ The BIG study reported that 60% of children with primary glaucoma and 28% with secondary glaucoma achieved IOP control (<21 mm Hg) at 1 year without additional medication.^4^

## SUMMARY

Uveitic glaucoma in children poses a significant risk of blindness and needs aggressive anti-inflammatory, antiglaucoma and amblyopia therapy to preserve vision. The uveitis specialist and glaucoma specialist need to work in close tandem, understanding that low IOP in uveitic children may herald a fresh attack of uveitis and that good control of uveitis may result in raised IOP due to trabecular scarring and/or abnormal corticosteroid response. Uveitic glaucoma in children continues to be a difficult problem to treat, and these children require extra care and judicious judgement to keep their IOP under control.
